# Wild Carrion Crows Choose Walnuts Based on Visual and Weight Cues

**DOI:** 10.1002/ece3.74554

**Published:** 2026-10-20

**Authors:** Torito Hamano, Yurika Mitsuhashi, Noriko Kondo, Masaki Shimada, Akitsugu Konno

**Affiliations:** ^1^ Department of Animal Sciences Teikyo University of Science Uenohara Yamanashi Japan; ^2^ Graduate School of Arts and Sciences The University of Tokyo Tokyo Japan; ^3^ Department of Animal Science and Biotechnology Azabu University Sagamihara Kanagawa Japan

**Keywords:** crows, decision‐making, discrimination, foraging behavior, physical cognition, walnuts

## Abstract

Assessment of the physical properties of concealed food items provides insight into the perceptual and cognitive mechanisms of animals. This study investigated the walnut selection behavior of wild carrion crows ( *Corvus corone* ) through two field experiments. In Experiment 1 (visual size preference), we presented walnuts with distinct visual sizes. Crows significantly preferred the larger walnut in both their first contact and final retrieval. In Experiment 2 (internal content preference), we presented walnuts of similar size but with different internal contents: filled or empty. While there was no preference for the first contact, crows significantly selected the filled walnut for final retrieval. The final selection was significantly influenced by the crows' handling behavior. In Experiment 2, the probability of switching was significantly higher when the first contact walnut was empty. Pre‐retrieval lifting behavior also played a distinct role in each experiment. In Experiment 1, lifting either the larger or smaller walnut increased the probability of it being ultimately retrieved, regardless of its size. In contrast, in Experiment 2, the number of pre‐retrieval lifts of the filled walnut significantly predicted its correct selection, whereas lifting the empty walnut had no such reinforcing effect on its retrieval. In both experiments, the probability of selecting the higher‐quality option increased with the log‐transformed weight ratio between the two walnuts, consistent with psychophysical principles of proportional weight discrimination. These findings demonstrate that wild crows flexibly integrate visual and weight information, utilizing active haptic sampling to compensate for unreliable visual cues and optimize foraging choices in a natural setting.

## Graphical abstract

This study investigated walnut selection in wild carrion crows ( *Corvus corone* ) through field experiments involving visual and weight‐based cues. Our results show that while crows initially prefer larger walnuts, they utilize active haptic sampling—such as switching and lifting—to accurately identify filled walnuts when visual cues are unreliable. The findings demonstrate that wild crows flexibly integrate multimodal information, with their weight discrimination performance aligning with psychophysical principles.

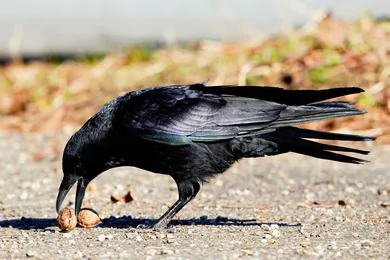

## 1 Introduction

Animals need to evaluate the quantity and quality of potential food items to maximize foraging efficiency (Pyke et al. 1977; Stephens and Krebs 1987). However, this evaluation is challenging for food items such as nuts or mollusks, where the edible portion is concealed by hard outer shells, preventing visual assessment of the internal state or volume. Furthermore, accessing these resources requires the acquisition of specialized cracking techniques. Indeed, various hard‐shell predators across diverse taxa have developed specialized behaviors to access protected nutrients, such as dropping nuts, seeds, or mollusks from heights, using stone tools to smash or hurl at hard‐shelled items, or exploiting artificial hazards like moving vehicles (Boesch and Boesch 1983; Fragaszy et al. 2004; Haslam et al. 2009; Nihei 1995; van Lawick‐Goodall and van Lawick‐Goodall 1966; Zach 1978). Since the technique for breaking these shells requires significant time and energy, the ability to select items with greater mass or content based on physical properties is expected to be favored by evolutionary processes (Cristol 1999; Maron 1982; Takagi and Ueda 2002; Uehara et al. 2024; Zach 1978).

The primary cue for animals to identify high‐energy food items is visual size, as a larger external volume generally predicts a greater quantity of edible content. Size‐selective foraging is well‐documented in non‐human animals. For instance, chimpanzees successfully choose larger food quantities by primarily responding to the total volume presented in a set (Beran et al. 2008). Similar laboratory studies have demonstrated that pigeons prefer larger grains and pellets (Killeen et al. 1993) and that Clark's Nutcrackers ( *Nucifraga columbiana* ) show a preference for cones with more viable seeds, larger quantities of seeds, and longer pine seeds (Christensen et al. 1991).

Food size preferences have also been observed in wild corvids. Northwestern crows ( *Corvus caurinus* ) prefer larger whelks to maximize energy return per drop (Zach 1978, 1979) and consistently select larger prey species regardless of differences in profitability (O'Brien et al. 2005). American crows ( *Corvus brachyrhynchos* ) have been shown to consume larger walnuts immediately while tending to cache smaller ones (Cristol 2001). Similarly, a preliminary study indicated that carrion crows in Japan prefer larger walnuts (Aoyama et al. 2017), suggesting a preference for visual volume. In this way, visual cues provide physical information available prior to contact, and are thus expected to guide the initial decision of which food item to approach (e.g., Heinrich et al. 1997).

The secondary cue for optimal foraging is weight, which is perceived through somatosensory input after making physical contact with the item. When edible portions are concealed by an outer shell, a discrepancy may arise between external size and internal content (e.g., Hadj‐Chikh et al. 1996). In such cases, weight perception becomes a reliable indicator of food quality. For instance, captive capuchin monkeys ( *Cebus apella* ) have been demonstrated to successfully learn to discriminate between filled and empty walnuts, primarily by relying on weight cues (Visalberghi and Neel 2003). In birds, wild chickadees ( *Poecile atricapillus* ) and red‐breasted nuthatches ( *Sitta canadensis* ) are known to selectively forage for filled sunflower seeds by discriminating weight differences (Heinrich et al. 1997). Similarly, studies on California scrub jays ( *Aphelocoma californica* ) and Mexican jays ( *Aphelocoma wollweberi* ) demonstrate that they can evaluate the quality of peanuts without opening the shells (Jablonski et al. 2015; Langen 1999). Through behaviors such as beak opening and closing, these jays select heavier and denser items, even when the peanuts are artificially weighted. These findings suggest that certain corvids can discriminate the presence of edible contents by using haptic (and acoustic) information to assess mass and density.

These findings collectively suggest that animals possess the ability to prefer food items with greater rewards by utilizing both visual size and weight information. However, little is known about how these two types of information are integrated or the specific sensory constraints governing such choices. It is generally established that perceptual discrimination depends on the ratio between stimuli rather than their absolute difference (Akre and Johnsen 2014; Eckert et al. 2018). While laboratory studies confirm that jungle crows ( *Corvus macrorhynchos* ) and carrion crows ( *Corvus corone* ) follow this principle in numerical judgments (Bogale et al. 2011; Ditz and Nieder 2016), research on the perception of visual size or weight remains scarce (Killeen et al. 1993; Moll and Nieder 2014). Furthermore, it is unclear how these cognitive mechanisms, typically measured in controlled settings, operate in complex natural environments. Field studies are therefore essential to determine whether such proportional discrimination facilitates adaptive decision‐making during wild foraging (Benson‐Amram et al. 2017). Examining cognitive performance and perceptual limitations in wild populations provides crucial insights into how sensory mechanisms govern adaptive behavioral strategies and drive the evolution of animal cognition (Morand‐Ferron et al. 2016).

The present study investigated how visual and weight information influence the walnut choices of carrion crows. We focused on multiple populations of wild crows that are frequently observed foraging on walnuts in their natural habitats. In Experiment 1, we presented crows with pairs of walnuts of different sizes (small vs. large). If the crows rely on visual cues, we predicted they would more frequently make initial contact with the larger walnut and ultimately retrieve it more often. In Experiment 2, we presented pairs of walnuts with nearly identical visual sizes but different contents (empty vs. filled). Since visual cues were uninformative, we predicted no preference in the initial contact but expected the crows to ultimately retrieve the filled walnuts more frequently by using weight as a cue.

Furthermore, we examined the crows' nut handling behaviors, specifically switching between walnuts within a pair and pre‐retrieval lifting, to understand how active information‐gathering contributes to their decision‐making process. We hypothesized that these behaviors would play a crucial role in evaluating internal properties when visual information is unreliable. In both experiments, we varied the weight ratios within the walnut pairs, predicting that the probability of selection would depend on the magnitude of this ratio, and that this relationship would be mediated by the crows' active handling of the items.

## 2 Materials and Methods

### 2.1 Study Sites and Subjects

Experiment 1 utilized four locations: two in Uenohara City, Yamanashi, Japan (the Katsura River riverbed [35.61671631598202, 139.11461204079305] and the Tokyo West Campus, Teikyo University of Science [35.6253925534876, 139.09710715780642]), and two in Sendai City, Miyagi, Japan (Tohoku University's Kawauchi North [38.25937285684766, 140.85224553959114] and Kawauchi South Campuses [38.25902745358657, 140.85405871279949]). Experiment 2 was conducted at the two Uenohara sites. The carrion crows at these sites are known to consume walnuts year‐round (Nihei 1995; Nihei and Higuchi 2001).

To preserve natural foraging contexts, we utilized a non‐invasive field methodology, avoiding capture or individual marking. Consequently, precise individual identification was not possible. However, based on our preliminary observations, the subjects at the Katsura River (Uenohara) likely consisted of mature individuals from a breeding pair, whereas those at the Tokyo West Campus were primarily groups of juveniles. Similarly, subjects at the Kawauchi North Campus (Sendai) appeared to be a breeding pair, while those at the South Campus were likely members of juvenile flocks. Because breeding pairs of carrion crows defend stable, exclusive territories, spatial overlap between the pair‐based sites was likely minimal. In contrast, given that juvenile corvids display high mobility and non‐territorial fission‐fusion dynamics (Jiguet and Gantin 2025), we cannot fully rule out the possibility of spatial overlap or dynamic movement of individuals between juvenile flock sites. Therefore, while our sampling across multiple geographically separated sites reduced the risk of sampling single local groups, repeated observations at the same locations inevitably lacked explicit individual identification. This likely resulted in a dynamic mix of data collection, where the same individuals were resampled over time alongside multiple individuals from different flock memberships continually replacing one another, representing an inherent limitation of our non‐invasive design. Similar field cafeteria experiments have been employed in previous studies to investigate foraging behavior in wild corvids (Chantoufi et al. 2025; Destrez et al. 2022).

### 2.2 Experiment 1: Walnut Size Preference (Small vs. Large)

We conducted field experiments on wild carrion crows to investigate their sensitivity to the physical characteristics of walnuts. Crows' nut‐choice behavior was assessed by presenting pairs of walnuts that differed in either size (Experiment 1) or content (Experiment 2) properties.

#### 2.2.1 Walnut Preparation

We used Japanese Walnuts ( *Juglans mandshurica* ) sourced from a single processor (Kobayashi Kazuo Shoten Co. Ltd.) in Nagano, Japan, to ensure morphological uniformity. For each nut, we measured height, width, and depth to the nearest 0.1 mm using a digital caliper. Estimated volume was calculated as the product of these dimensions. Each nut was weighed to the nearest 0.1 g. Given the high positive correlation between estimated volume and weight (*r* = 0.81, *p* < 0.001), weight was utilized as the primary metric for walnut size.

Pairs consisted of one “Small” walnut (mean ± SD: 7.38 ± 1.16 g; range: 4.10–9.80 g) and one “Large” walnut (9.23 ± 1.05 g; range: 6.70–12.10 g). The mean weight difference within pairs was 1.85 ± 0.98 g (range: 0.00 to 4.60 g). The mean mass ratio (large walnut/small walnut) was 1.27 ± 0.20 (range: 1.00–2.19). For statistical analysis, we used the log‐transformed weight ratio (Weight Ratio [log]; large walnut/small walnut), which ranged from 0.00 to 0.79 (mean = 0.23 ± 0.14). Walnut pairs (*N* = 4) with no weight difference (0.00 g) were excluded from our GLMs.

#### 2.2.2 Field Procedure

A pair of walnuts was placed in an open area, separated by less than 30 cm. The spatial position (left or right) of the larger walnut was pseudo‐randomized (Fellows 1967). A trial began upon placement and ended when a crow retrieved one of the walnuts. The experimenter immediately retrieved the remaining nut. A total of 241 successful trials were analyzed. The experiment was conducted between October 31, 2021, and July 2, 2022. An example of the field experiment is shown in Figure S1.

#### 2.2.3 Behavioral Coding

All trials were recorded using a digital video camera (HC‐V480M, Panasonic), and for each trial, we coded variables to evaluate the sequential decision‐making process of the crows. We first identified the First Contact Walnut (the nut first touched by the crow; coded as 1 = large, 0 = small) to assess initial visual preference, followed by the Finally Retrieved Walnut (the nut ultimately carried away; 1 = large, 0 = small) to indicate the final choice.

To quantify the evaluative process occurring between this initial contact and the final choice, we recorded occurrence of Switching Behavior, which included both a binary measure of whether the crow touched both nuts (1 = yes, 0 = no). Furthermore, we recorded the Number of Pre‐retrieval Lifts separately for the large and small walnuts (for statistical analysis, the final lifting act leading to retrieval was excluded to evaluate only the evaluative lifting behavior prior to the final choice). Finally, Retrieval Latency was measured from the moment of landing to taking flight with the chosen nut (to the nearest 0.1 s) to serve as an index of the time investment required for discrimination and choice.

#### 2.2.4 Statistical Analysis

We used Generalized Linear Models (GLMs) with a binomial distribution and logit link for all behavioral predictions. To account for the non‐linear nature of sensory perception, the weight ratio of the two walnuts was log‐transformed (Weight Ratio [log]). To facilitate model convergence and facilitate the comparison of effect sizes, all continuous predictor variables were z‐transformed (standardized) prior to analysis.

##### 2.2.4.1 First Contact

To determine whether crows could visually discriminate between walnuts prior to physical contact, we set First Contact Walnut (1 = large, 0 = small) as the response variable. We first fitted an intercept‐only GLM to test whether the probability of first contact differed from chance. We then constructed a Full GLM model including the Walnut Weight Ratio (log), Trial Order, and Site (University [Uenohara], River [Uenohara], North Campus [Sendai], and South Campus [Sendai], reference: University) as fixed predictors.

##### 2.2.4.2 Final Retrieval

For the analysis of the ultimate choice, Finally Retrieved Walnut (1 = large, 0 = small) was used as the response variable. Fixed predictors included the Walnut Weight Ratio (log), Small Walnut Weight (the absolute weight of the smaller walnut), Switching Behavior (1 = yes, 0 = no), the Number of Pre‐retrieval Lifts for each walnut type, Retrieval Latency, Trial Order, and Site.

To assess multicollinearity among explanatory variables, Generalized Variance Inflation Factors (GVIFs) were calculated for all GLMs in Experiment 1. Adjusted VIF values across all models ranged from 1.02 to 1.75, confirming the absence of significant multicollinearity.

### 2.3 Experiment 2: Walnut Content Preference (Empty vs. Filled)

#### 2.3.1 Walnut Preparation

To manipulate content while maintaining uniform external morphology, we used Shinano Walnuts (*Juglans regia L*.) cultivated for consumption. Pairs were selected based on similar estimated volumes (maximum difference < 1500 mm^3^).

Nuts were processed into either “Empty” (kernel removed) or “Filled” (intact kernel) stimuli (Figure S2). Shells were soaked for 24 h, heat‐dried, and carefully cracked along the natural seam. For Empty walnuts, the cotyledon was removed before rejoining the halves with cyanoacrylate adhesive. Filled walnuts underwent the same cracking and rejoining procedure but retained their kernels. Filled walnuts was 9.93 ± 1.48 g (range: 6.50–14.10 g), while Empty walnuts averaged 5.72 ± 1.01 g (range: 3.00–7.60 g). The mass ratio (filled walnut/empty walnut) was 1.78 ± 0.32 (range: 1.07–3.33). For statistical analysis, we calculated the log‐transformed weight ratio, which was 0.56 ± 0.20 (range: 0.07–1.20). Twenty‐four pairs were excluded from statistical modeling due to missing walnut weight data.

#### 2.3.2 Field Procedure

The experimental protocol followed that of Experiment 1, with 80 successful trials analyzed (40 per site in Uenohara). The experiment at the Katsura River site was conducted from June 22, 2021, to December 3, 2021, and the experiment at the University site ran from January 8, 2021, to October 29, 2021.

#### 2.3.3 Behavioral Coding

The behavioral coding method was identical to Experiment 1. First Contact Walnut and Finally Retrieved Walnut were coded as binary variables (1 = filled, 0 = empty). We recorded Switching Behavior (1 = yes, 0 = no), the Number of Pre‐retrieval Lifts separately for Filled and Empty walnuts, and Retrieval Latency.

#### 2.3.4 Statistical Analysis

We used Generalized Linear Models (GLMs) and Firth's penalized logistic regression models using a binomial distribution with logit link function. As in Experiment 1, all continuous predictors were *z*‐transformed prior to model fitting.

##### 2.3.4.1 First Contact

To determine whether crows could visually discriminate between walnuts prior to physical contact, we set First Contact Walnut (1 = filled, 0 = empty) as the response variable. We first fitted an intercept‐only GLM to test whether the probability of first contact differed from chance. We then constructed a Full GLM model including the Walnut Weight Ratio (log), Trial Order, and Site (University [Uenohara], River [Uenohara]; reference: University) as fixed predictors.

##### 2.3.4.2 Final Retrieval

For the analysis of the ultimate choice, Firth's penalized logistic regression was employed (using the logistf package in R) to account for potential small sample size bias and complete separation. Finally Retrieved Walnut (1 = filled, 0 = empty) was used as the response variable. Fixed predictors included the Walnut Weight Ratio (log), Empty Walnut Weight (the absolute weight of the empty walnut), Switching Behavior (1 = yes, 0 = no), Pre‐retrieval Lifts of filled walnuts, Pre‐retrieval Lifts of empty walnuts, Retrieval Latency, Trial Order, and Site.

##### 2.3.4.3 First Contact and Switching Behavior

To analyze the decision‐making process in Experiment 2, we set the occurrence of Switching Behavior (1 = yes, 0 = no) as the response variable in a GLM. Fixed predictors were the property of the First Contact Walnut (Empty vs. Filled; reference: Empty), Trial Order, and Site. This model tested whether the property of the first walnut touched influenced the subsequent probability of switching. To assess multicollinearity among explanatory variables, Variance Inflation Factors (VIFs) were calculated for all GLMs. VIF values across all models ranged from 1.02 to 3.45 for Experiment 2, confirming the absence of significant multicollinearity.

### 2.4 Effect Size and Interpretation

For all models, we reported standardized coefficients (β), standard errors (SE), *z* values or chi‐squared statistics, and *p* values. To evaluate the practical significance of the predictors, odds ratios (OR) and their 95% confidence intervals (CI) were calculated. All statistical analyses were performed using R (version 4.5.2).

## 3 Results

### 3.1 Experiment 1: Walnut Size Preference (Small vs. Large)

#### 3.1.1 First Contact

In 66.8% of trials, the first contact was made with the larger walnut (161 out of 241 trials; Figure 1; Table S1). An intercept‐only GLM (logLik = −149.43, AIC = 300.86) showed that the probability of the first contact being with the larger walnut was significantly higher than chance (Estimate = 0.731, *z* = 5.273, *p* < 0.001).

**FIGURE 1:**
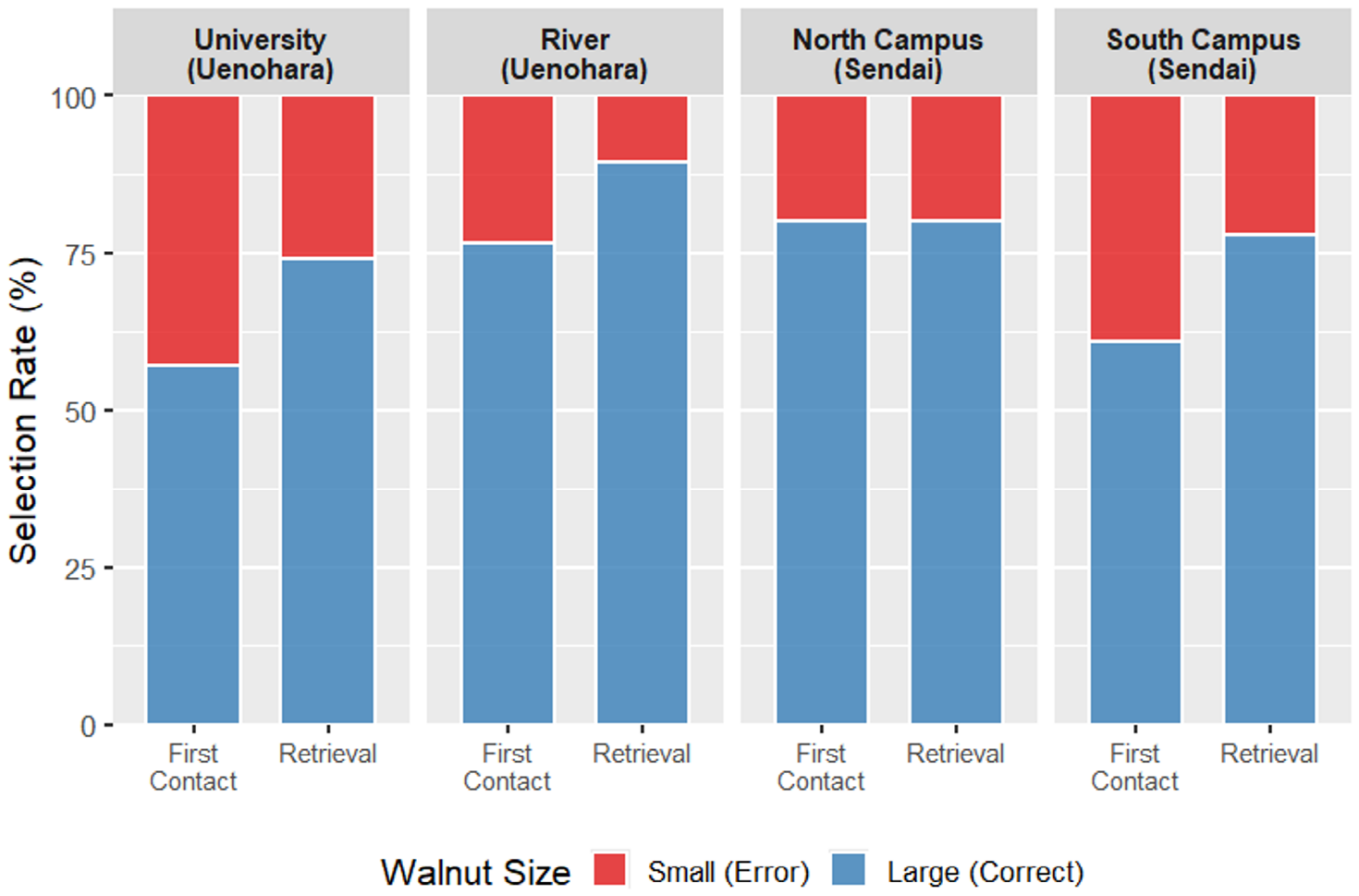
Summary of walnut selection across four study sites in Experiment 1 (size preference).

The full GLM (logLik = −134.60, AIC = 281.19; Table 1) revealed that the probability of targeting the larger walnut first was positively correlated with the weight ratio (Estimate = 0.688, *z* = 4.130, *p* < 0.001). For every one‐standard‐deviation increase in the log ratio, the odds of selecting the larger walnut first increased by 1.99 times (95% CI: 1.446–2.798).

**TABLE 1:** Estimated coefficients of GLMs predicting the first contact of the larger walnut in Experiment 1 (size preference).

Model	Term	*β*	SE	*Z*	*p*	OR	95% CI
Intercept‐only	**Intercept**	**0.731**	**0.139**	**5.273**	**< 0.001**	**2.078**	**1.582–2.730**
Full model	Intercept	0.258	0.369	0.698	0.485	1.294	0.629–2.688
	**Weight Ratio (log)**	**0.688**	**0.167**	**4.130**	**< 0.001**	**1.991**	**1.446–2.798**
	Trial Order	0.001	0.007	0.189	0.850	1.001	0.988–1.015
	**Site (River)**	**1.040**	**0.365**	**2.844**	**0.004**	**2.828**	**1.396–5.882**
	**Site (North Campus)**	**1.516**	**0.723**	**2.097**	**0.036**	**4.554**	**1.218–22.459**
	Site (South Campus)	0.218	0.382	0.571	0.568	1.244	0.590–2.651

*Note:* *N* = 237 (4 trials with zero weight difference were excluded). The dependent variable is First Contact (binary: 1 = larger walnut contacted first, 0 = smaller walnut contacted first). Predictors in Full Model include Weight Ratio (log), Trial Order, and Site (reference: University) as fixed effects. Weight Ratio was standardized (*z*‐transformed) prior to analysis. Intercept‐only: logLik = −149.43, AIC = 300.86; Full Model: logLik = −134.60, AIC = 281.19. Odds ratios and 95% confidence intervals (CI) were calculated using the Wald method. Significant effects (*p* < 0.05) are shown in bold.

Trial order had no significant effect on the probability of first contact with the larger walnut (Estimate = 0.001, *z* = 0.189, *p* = 0.850). In contrast, study site significantly affects the initial choice (Type III ANOVA = 12.35, df = 3, *p* = 0.006). Compared to the baseline site (University), crows at the River site (Estimate = 1.040, *z* = 2.844, *p* = 0.004; OR = 2.828, 95% CI: 1.398–5.885) and North Campus (Estimate = 1.516, *z* = 2.097, *p* = 0.036; OR = 4.554, 95% CI: 1.222–22.502) were significantly more likely to make first contact with the larger walnut, whereas no significant difference was observed for South Campus (Estimate = 0.218, *z* = 0.571, *p* = 0.568; OR = 1.244, 95% CI: 0.590–2.652; Table 1).

#### 3.1.2 Final Retrieval

The final selection rate for the larger walnut was 80.9% (195 out of 241 trials; Figure 1, Table S1). Selection rates across study sites were 74.0% (Uenohara University), 89.4% (Uenohara River), 80.0% (Sendai North Campus), and 78.0% (Sendai South Campus).

Before making the final selection, the crows engaged in nut handling behaviors. Switching behavior between the two walnuts occurred in 48.1% of the trials (116 out of 241 trials), with a mean frequency of 1.65 ± 2.65 (range: 0–18) switches. The crows performed a mean of 1.23 ± 1.60 (range: 0–10) pre‐retrieval lifts on the larger walnut, whereas the number of pre‐retrieval lifts on the smaller walnut was lower (0.31 ± 1.86; range = 0–5). The mean retrieval latency, from the first contact to the final retrieval, was 14.61 ± 1.86 s (range: 0.70–78.32 s).

The full GLM included the log‐transformed weight ratio, small walnut weight, switching frequency, the number of pre‐retrieval lifts of both larger and smaller walnuts, retrieval latency, trial order, and study site (Table 2). This GLM (LogLik = −60.57, AIC = 143.13) revealed that the log‐transformed weight ratio had a highly significant positive effect on the probability of choosing the larger walnut (Estimate = 1.484, *z* = 3.712, *p* < 0.001; Table 2, Figure 2). For every one‐standard‐deviation increase in the log‐ratio, the odds of selecting the larger walnut increased by 4.411 times (95% CI: 2.118–10.203).

**TABLE 2:** Estimated coefficients of the GLM predicting the final retrieval of the larger walnut in Experiment 1 (size preference).

	*β*	SE	*Z*	*p*	OR	95% CI
**Intercept**	**1.522**	**0.712**	**2.139**	**0.032**	**4.580**	**1.208–20.354**
**Weight Ratio (log)**	**1.484**	**0.400**	**3.712**	**< 0.001**	**4.411**	**2.118–10.203**
Small Nut Weight	0.561	0.353	1.590	0.112	1.753	0.901–3.642
Switching (yes)	0.438	0.700	0.625	0.532	1.549	0.405–6.626
Retrieval Latency	0.806	0.533	1.513	0.130	2.239	0.858–6.969
**Pre‐Lifts (Large Nut)**	**1.602**	**0.603**	**2.658**	**0.008**	**4.963**	**1.718–19.117**
**Pre‐Lifts (Small Nut)**	**−1.605**	**0.358**	**−4.487**	**< 0.001**	**0.201**	**0.093–0.384**
Trial Order	0.002	0.011	0.139	0.889	1.002	0.979–1.025
Site (River)	2.058	0.714	2.882	0.004	7.834	2.058–34.931
Site (North Campus)	0.136	1.208	0.113	0.910	1.146	0.112–14.041
**Site (South Campus)**	**1.414**	**0.705**	**2.007**	**0.045**	**4.113**	**1.082–17.625**

*Note:* *N* = 236 (one missing observation was removed; 4 trials with zero weight difference were excluded). The dependent variable is Final Retrieval (binary: 1 = larger walnut, 0 = smaller walnut). Predictors (Weight Ratio, Small Nut Weight, Retrieval Latency, Pre‐Lifts [Large Nut], and Pre‐Lifts [Small Nut]) were standardized (z‐transformed) prior to analysis. Switching Behavior (binary: y = yes, *n* = no; reference = *n*), Trial Order and Study Site (reference category: University) were also included as fixed effects. Log‐likelihood = −60.565, AIC = 143.130. Odds ratios and 95% confidence intervals (CI) were calculated using the Wald method. Significant effects (*p* < 0.05) are shown in bold.

**FIGURE 2:**
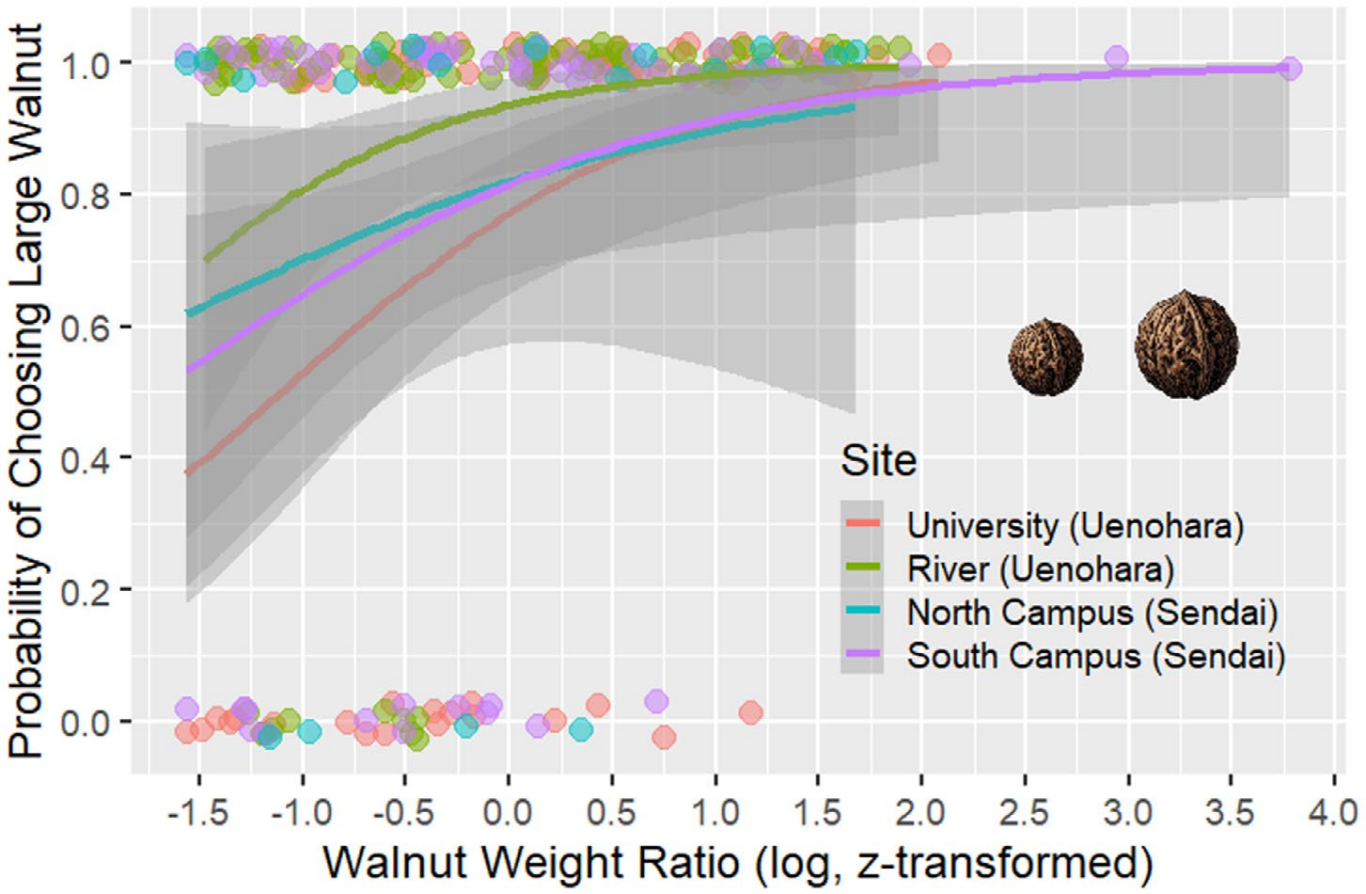
The effect of weight ratio on the probability of larger walnut selection by crows in Experiment 1 (size preference). The curves represent predicted probabilities from logistic regression for each study site. Dots (*N* = 237) represent individual trials (jittered for visibility). Shaded areas indicate 95% confidence intervals.

The crows' behavior significantly influenced their preferences (Table 2). Both switching behavior (yes/no) (Estimate = 0.438, *z* = 0.625, *p* = 0.532; OR = 1.549, 95% CI: 0.405–6.626) and retrieval latency (Estimate = 0.806, *z* = 1.513, *p* = 0.130; OR = 2.239, 95% CI: 0.858–6.969) showed positive effects on the choice of the larger walnut, though neither reached statistical significance. Pre‐retrieval lifts of the larger walnut had a significant positive effect on the probability of choosing it (Estimate = 1.602, *z* = 2.658, *p* = 0.008; OR = 4.963, 95% CI: 1.718–19.117), whereas pre‐retrieval lifts of the smaller walnut had a negative effect on the likelihood of selecting the larger one (Estimate = −1.605, *z* = −4.487, *p* < 0.001; OR = 0.201, 95% CI: 0.093–0.384).

Trial order had no significant effect on final retrieval (Estimate = 0.002, *z* = 0.139, *p* = 0.889; OR = 1.002, 95% CI: 0.979–1.025). In contrast, study site significantly influenced final retrieval choice (Type III ANOVA, Chisq = 10.963, df = 3, *p* = 0.012). Compared to the baseline site (University), crows at the River site (Estimate = 2.058, *z* = 2.882, *p* = 0.004; OR = 7.834, 95% CI: 2.058–34.931) and South Campus site (Estimate = 1.414, *z* = 2.007, *p* = 0.045; OR = 4.113, 95% CI: 1.082–17.625) showed a significantly higher likelihood of retrieving the larger walnut, whereas no significant difference was observed for North Campus (Estimate = 0.136, *z* = 0.113, *p* = 0.910; OR = 1.146, 95% CI: 0.112–14.041).

### 3.2 Experiment 2: Walnut Content Preference (Empty vs. Filled)

#### 3.2.1 First Contact

In 58.8% of trials, the first contact was made with the filled walnut (47 out of 80 trials; Figure 3, Table S2, Table 3). An intercept‐only GLM showed that this probability was not significantly different from chance (Estimate = 0.215, *z* = 0.800, *p* = 0.424). The initial choice was not significantly affected by the log‐transformed weight ratio (Estimate = 0.308, *z* = 1.013, *p* = 0.311). None of the other predictors in the full model had a significant effect on the first contact, including trial order (Estimate = 0.046, *z* = 1.475, *p* = 0.140) and study site (Estimate = 0.879, *z* = 1.285, *p* = 0.199).

**FIGURE 3:**
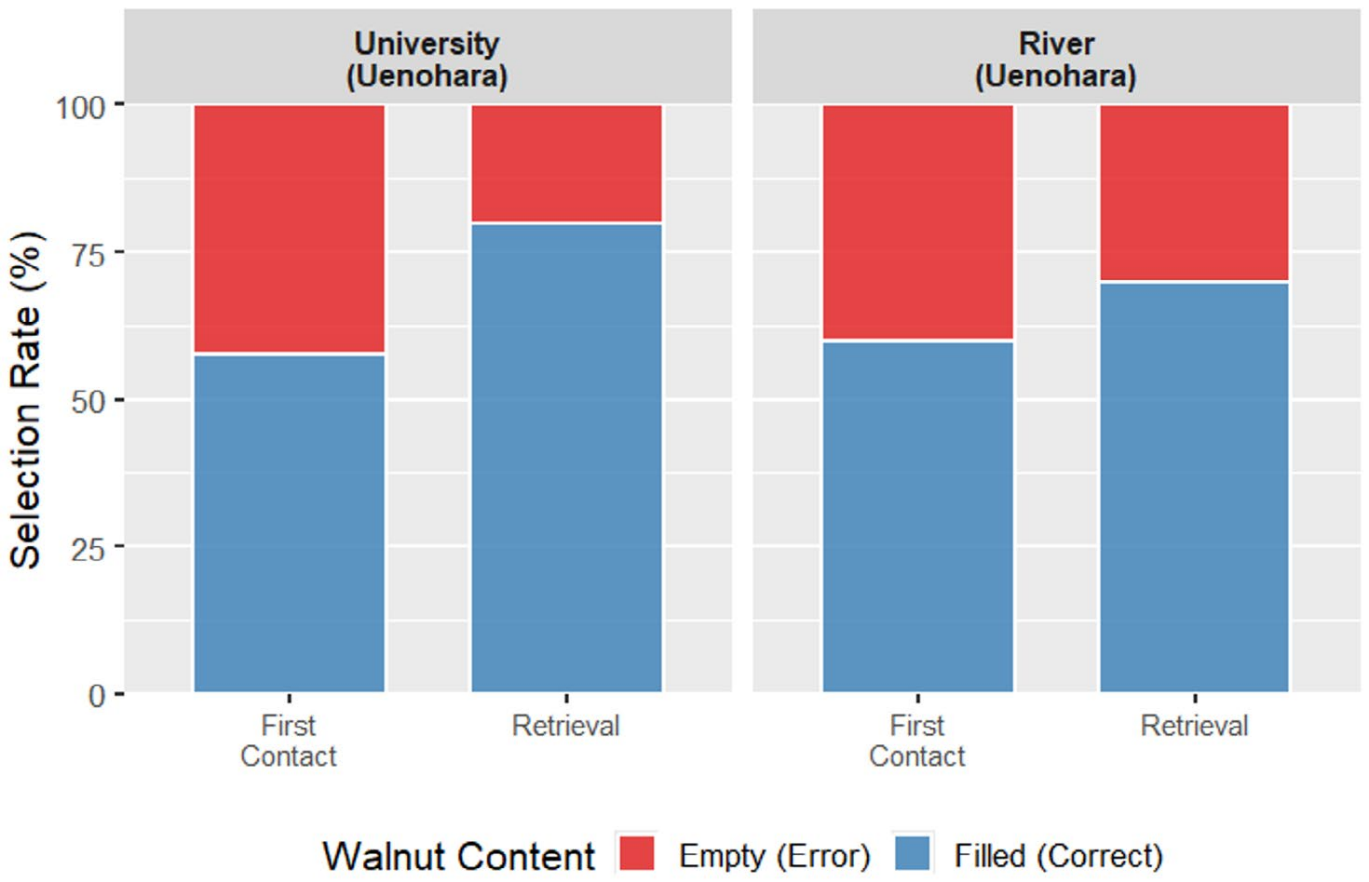
Summary of walnut selection across two study sites in Experiment 2 (content preference).

**TABLE 3:** Estimated coefficients of the GLMs predicting the first walnut touched in Experiment 2 (content preference).

Model	Term	*β*	SE	*Z*	*p*	OR	95% CI
Intercept‐only	Intercept	0.215	0.269	0.800	0.424	1.240	0.734–2.124
Full Model	Intercept	−1.451	1.072	−1.354	0.176	0.234	0.026–1.832
	Weight Ratio (log)	0.308	0.304	1.013	0.311	1.361	0.764–2.573
	Trial Order	0.046	0.031	1.475	0.140	1.047	0.987–1.124
	Site (River)	0.879	0.684	1.285	0.199	2.409	0.645–9.729

*Note:* *N* = 56 for both models (24 trials were removed due to missing walnut weight data). The dependent variable is First Contact (binary: 1 = filled walnut contacted first, 0 = empty walnut contacted first). Predictors in Full Model include Weight Ratio (log), Trial Order, and Site (reference category: University) as fixed effects. Weight Ratio (log) was standardized (z‐transformed) for the analysis. Intercept‐only: logLik = −38.49, AICc = 79.06. Full Model: logLik = −36.33, AICc = 81.44. Odds ratios and 95% confidence intervals (CI) were calculated using the Wald method. Significant effects (*p* < 0.05) are shown in bold.

#### 3.2.2 Final Retrieval

The final selection accuracy for the filled walnut was 75.0% (60 out of 80 trials; Figure 3, Table S2). Accuracy rates were 70.0% at the University site (28 out of 40 trials) and 80.0% (32 out of 40 trials) at the River study sites.

During the nut handling process, switching behavior between the two walnuts occurred in 45.0% of the trials (36 out of 80 trials), with a mean frequency of 1.13 ± 1.69; range: 0–7 switches. On average, the crows performed 0.83 ± 1.33 (range: 0–7) pre‐retrieval lifts on the filled walnut and 0.26 ± 0.90 (range: 0–5) lifts on the empty walnut. The mean retrieval latency in this experiment was 11.38 ± 11.72 s (range: 1.00–49.00 s).

Firth's penalized logistic regression revealed that the log‐transformed weight ratio had a significant positive effect on the probability of choosing the filled walnut (Estimate = 1.202, Chisq = 4.749 *p* = 0.029; Table 4, Figure 4). For every one‐standard‐deviation increase in the log‐ratio, the odds of selecting the filled walnut increased by 3.326 times (95% CI: 1.118–17.783).

**TABLE 4:** Estimated coefficients of the Firth's penalized logistic regression model for predicting the final retrieval of the filled walnut in Experiment 2 (Content Preference).

	*β*	SE	Chisq	*p*	OR	95% CI
Intercept	0.611	1.558	0.122	0.727	1.842	0.049–62.818
**Weight Ratio (log)**	**1.202**	**0.573**	**4.749**	**0.029**	**3.326**	**1.118–17.783**
Empty Nut Weight	−0.411	0.627	0.359	0.549	0.663	0.145–2.494
Switching (yes)	1.220	1.248	0.760	0.383	3.387	0.223–83.933
Retrieval Latency	0.388	0.704	0.246	0.620	1.474	0.360–14.612
**Pre‐lifts (Filled)**	**1.861**	**0.965**	**4.793**	**0.029**	**6.427**	**1.118–126.498**
Pre‐lifts (Empty)	0.111	0.273	0.146	0.702	1.117	0.617–3.261
Trial Order	0.037	0.044	0.612	0.434	1.037	0.948–1.153
Site (River)	0.303	1.208	0.052	0.820	1.354	0.096–22.084

*Note:* *N* = 54 (24 trials with missing weight data and 2 trials with missing retrieval latency data were removed). The dependent variable is Final Retrieval (binary: 1 = filled walnut, 0 = empty walnut). Predictors (Weight Ratio, Empty Nut Weight, Retrieval Latency, Pre‐Lifts [Filled Nut], and Pre‐Lifts [Empty Nut]) were standardized (*z*‐transformed) prior to analysis. Switching Behavior (binary: y = yes, *n* = no; reference = *n*), Trial Order and Study Site (reference: University) were also included as fixed effects. Model Likelihood Ratio Test: Chisq = 16.72, df = 8, *p* = 0.030. Chi‐square statistics, Odds ratios (OR) and 95% confidence intervals (CI) were calculated using Firth's penalized logistic regression. Significant effects (*p* < 0.05) are shown in bold.

**FIGURE 4:**
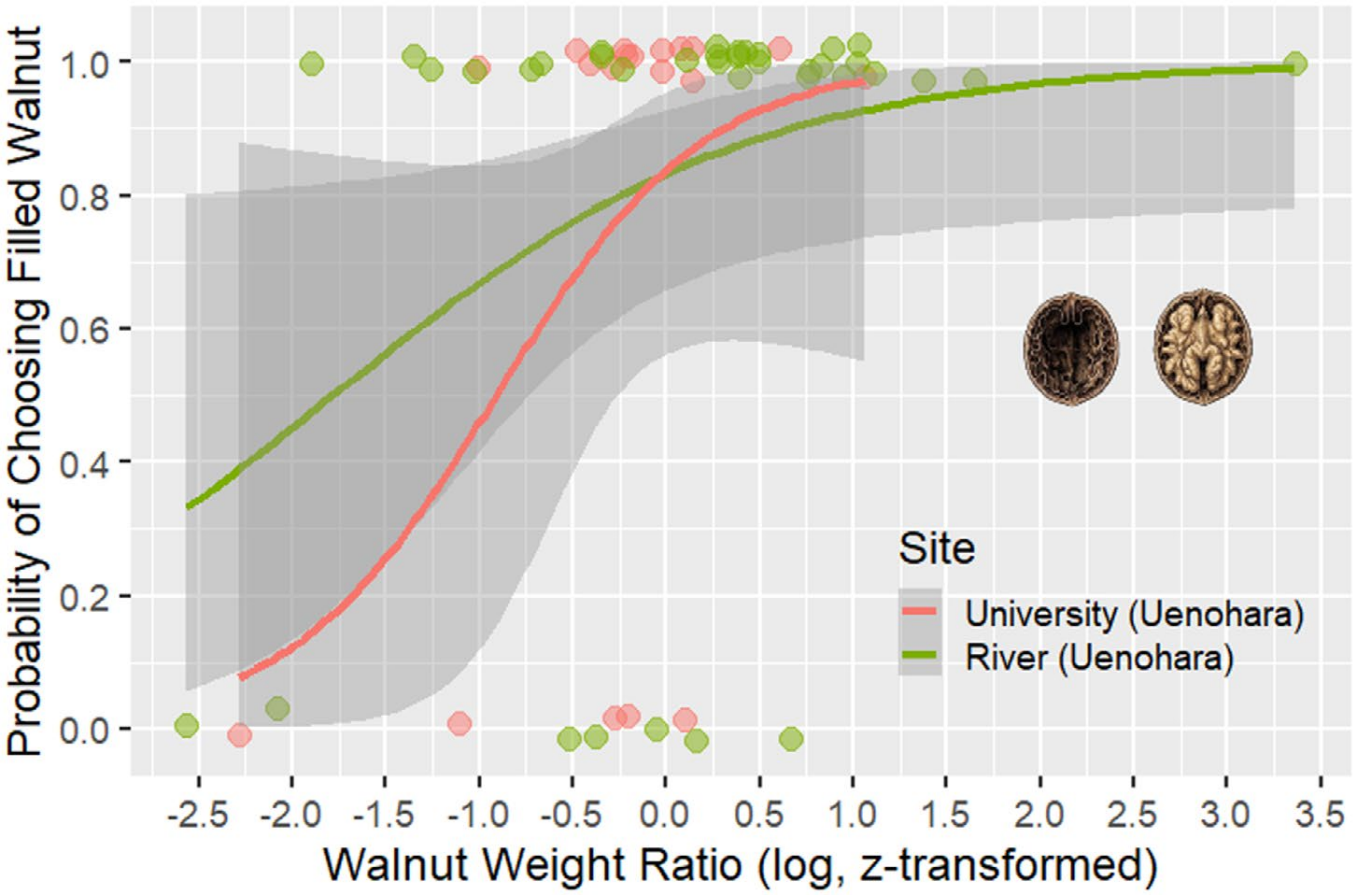
The effect of weight ratio on the probability of filled walnut selection by crows in Experiment 2 (content preference). The curves represent predicted probabilities from logistic regression for each study site. Dots (*N* = 56) represent individual trials (jittered for visibility). Shaded areas indicate 95% confidence intervals.

The crows' behavior significantly influenced their preferences for the filled walnut (Table 4). Switching behavior showed a positive association with selecting the filled walnut (OR = 3.387), but this effect did not reach statistical significance (Estimate = 1.200, Chisq = 0.760, *p* = 0.383). The number of pre‐retrieval lifts of filled walnuts also had a significant positive effect on the probability of choosing it (Estimate = 1.861, Chisq = 4.793, *p* = 0.029). For each standard‐deviation increase in pre‐retrieval lifts on the filled walnut, the odds of selecting the filled walnut increased by 6.427 times (95% CI: 1.118–126.498). None of the other variables (i.e., empty nut weight, pre‐retrieval lifts on empty nuts, retrieval latency, trial order, and study site) had a significant effect on final retrieval (Table 4).

#### 3.2.3 First Contact and Switching Behavior

When the first walnut touched was empty, switching behavior occurred in 60.6% of trials (20 out of 33 trials), whereas when the first contact was made with a filled walnut, switching occurred in only 34.0% of trials (16 out of 47 trials).

The occurrence of nut‐switching behavior was significantly influenced by the content of the first walnut touched (Estimate = 1.400, *z* = 2.660, *p* = 0.008; Table 5). When the first walnut touched was empty, the odds of switching to the alternative walnut were 4.053 times higher (95% CI: 1.494–11.949) than when the first contact was made with a filled walnut (Figure 5).

**TABLE 5:** Estimated coefficients of the GLM predicting switching behavior between walnuts in Experiment 2 (Content Preference).

	*β*	SE	*Z*	*p*	OR	95% CI
**Intercept (Filled)**	**−2.602**	**0.738**	**−3.528**	**< 0.001**	**0.074**	**0.015–0.283**
**First Contact: Empty**	**1.400**	**0.526**	**2.660**	**0.008**	**4.053**	**1.494–11.949**
**Trial Order**	**0.067**	**0.023**	**2.888**	**0.004**	**1.069**	**1.024–1.121**
Site (River)	0.783	0.510	1.536	0.125	2.187	0.819–6.133

*Note:* *N* = 80. The dependent variable is Switching Behavior (binary: 1 = switched between walnuts, 0 = did not switch). Predictors include First Contact (reference category: Filled), Trial Order, and Study Site (reference: University) as fixed effects. Log‐likelihood = −46.49. ALCc = 101.51. Odds ratios (OR) and 95% confidence intervals (CI) were calculated using the Wald method. Significant effects (*p* < 0.05) are shown in bold.

**FIGURE 5:**
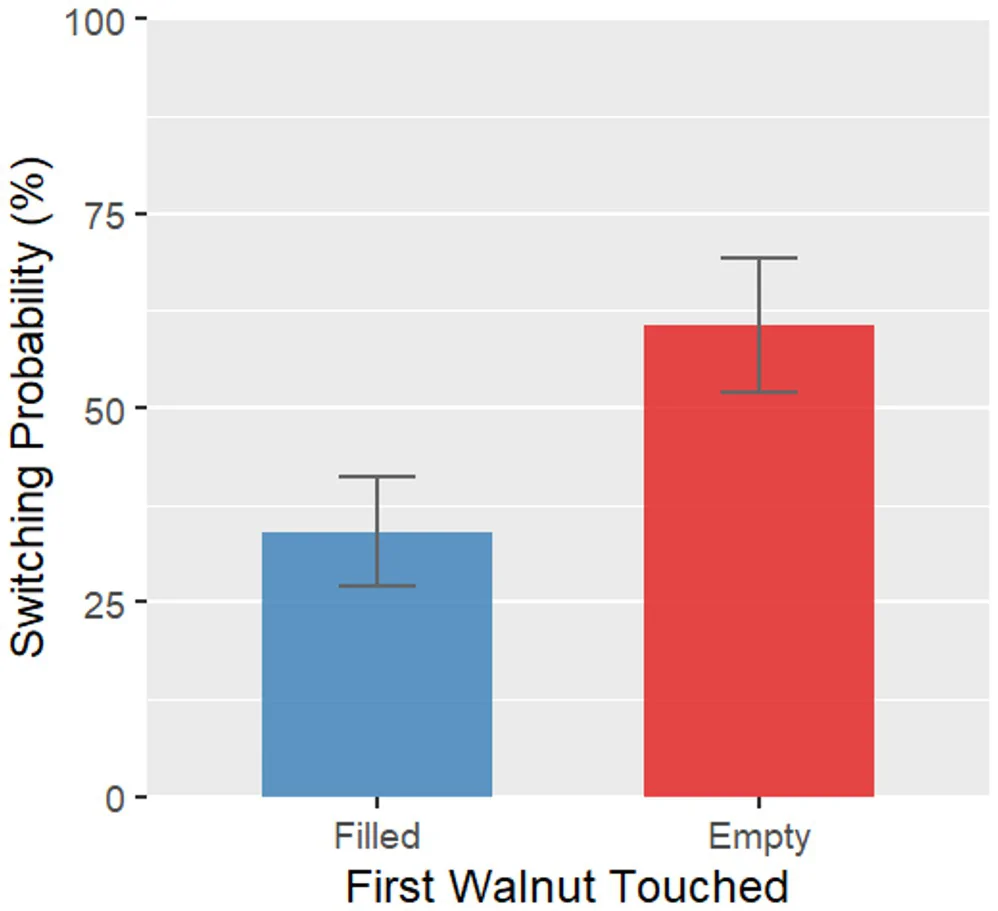
Probability of switching behavior based on initial tactile assessment in Experiment 2. The bar graph represents the mean percentage of trials in which crows switched to the alternative walnut after their first contact. Crows were significantly more likely to switch when the walnut touched first was empty (red bar) compared to when it was filled (blue bar). Error bars represent the standard error (SE). *N* = 80 trials.

In addition, trial order had a significant positive effect on switching behavior (Estimate = 0.067, *z* = 2.888, *p* = 0.004). For each additional trial, the odds of switching between walnuts increased by 1.069 times (95% CI: 1.024–1.121). Study site did not significantly affect switching behavior (Estimate = 0.783, *z* = 1.536, *p* = 0.125).

## 4 Discussion

One of the primary findings of this study is that wild carrion crows prioritize visual information as the initial cue for prey selection. The choice of which walnut the crow approached first depended on the difference in the visual dimensions of the prey. In Experiment 1, when presented with walnut pairs of different sizes, the probability of the crows making initial contact with the larger walnut was significantly higher than for the smaller one. In contrast, in Experiment 2, where walnut pairs were of nearly identical size but differed in content, there was no significant difference in which item the crows first touched. These findings suggest that the initial decision‐making in crow foraging is primarily driven by a preference for larger‐looking prey.

Avian species are generally considered a visually dominant taxon, with their foraging behavior relying on sensitive visual cognitive systems (Martin 2012; Niu et al. 2022). The visual dominance of crows in prey selection observed in this study is consistent with previous research on congeneric species. In American crows, walnuts selected for caching were significantly smaller than those eaten on‐site, indicating that they discriminate prey based on visual size to suit different foraging strategies (Cristol 2001). Similarly, Northwestern crows have been shown to preferentially select larger prey species, even when such choices do not necessarily correlate with increased foraging efficiency or net energy gain (O'Brien et al. 2005). The results of the present study corroborate the idea that wild corvids prioritize visual cues when making foraging decisions (Chantoufi et al. 2025; Destrez et al. 2022), maintaining this visual‐first approach even when utilizing food resources where the edible parts are concealed.

Building on this initial visual bias, our second primary finding demonstrates that crows subsequently refine their choices, suggesting a sequential decision‐making process. In Experiment 1, the accuracy of the final retrieval was even higher than that of the initial contact, suggesting that visual information was reinforced by additional sensory input. In Experiment 2, while initial contact was at chance levels due to the lack of visual cues, the probability of ultimately retrieving the filled walnut was significantly higher than for the empty one. These findings suggest that walnut selection is not a singular event but a sequential evaluation, where initial visual heuristics are cross‐validated through subsequent haptic assessment.

This refined assessment of prey quality is likely mediated by bill‐mediated handling behaviors prior to final choice. While the binary occurrence of switching behavior (interacting with both items) did not directly reach statistical significance as a main effect predictor for final retrieval in either experiment, it played a conditional role in information gathering. The function of the occurrence of switching was particularly striking in Experiment 2, where visual cues were entirely uninformative. When the first walnut touched was empty, the odds of switching to the alternative walnut were approximately four times higher than when the first contact was made with a filled walnut. This behavioral sequence suggests that haptic feedback from an empty walnut, specifically a mismatch between the expected and perceived sensory input, prompts the crow to abandon the current item and move to the alternative. Additionally, trial order significantly increased the likelihood of switching in Experiment 2, suggesting that experience within the experimental context enhanced the crows' tendency to cross‐check options.

However, the precise nature of the sensory information obtained through bill contact remains unclear. For instance, capuchin monkeys utilize finger‐tapping behaviors to improve their discrimination accuracy regarding nut contents (Visalberghi and Neel 2003), while jays employ rapid bill‐snapping to reject empty or deceptive peanuts (Jablonski et al. 2015). These specialized behaviors are thought to function by utilizing not only haptic feedback but also auditory feedback to enhance the accuracy of discriminating the volume and density of hidden food items. Since we cannot exclude the possibility that the crows in our study similarly utilized acoustic cues during handling, further research employing high‐speed videography and acoustic analysis is necessary to identify the fine‐scale sensory inputs that drive these sophisticated foraging decisions.

Pre‐retrieval lifting behavior also contributed significantly to the crows' final choice of walnuts, though its function appeared to differ between the two experiments. The act of grasping and lifting an item with the bill is thought to facilitate weight perception by generating inertial information through active movement (Jablonski et al. 2015). In Experiment 1, lifting any walnut consistently increased its likelihood of retrieval, suggesting a general reinforcement of choice regardless of size. In contrast, in Experiment 2, only the lifting of filled walnuts served as a significant predictor of the final selection, whereas lifting empty items had no such reinforcing effect. This discrepancy suggests that in the size‐discrimination task (Experiment 1), the haptic sensation of weight gained through lifting serves as a form of perceptual validation to confirm the presence of content, effectively reinforcing a choice already biased by visual size. In the content‐discrimination task (Experiment 2), however, where visual cues are identical, lifting serves as a critical mechanism for evaluating the correspondence between observed size and expected mass.

In natural settings, it is unusual for a walnut's internal contents to be removed while the shell remains intact. When handling such artificial stimuli, the lifting action likely allows crows to detect a “prediction error”, for instance, when a walnut is significantly lighter than its size suggests. This interpretation is consistent with research using expectation‐of‐violation paradigms, which has indicated that rooks and Eurasian jays possess an understanding of support relations, specifically, that objects will fall if they are not adequately supported by a solid surface (Bird and Emery 2010; Davidson et al. 2017). Furthermore, New Caledonian crows have been shown to infer the weight of novel objects based on their movement (Jelbert et al. 2019). These findings suggest that crows possess an ability to form mental representations of an object's mass. Although further validation is required, our results provide preliminary evidence suggesting that when a crow lifts a walnut, it may not be merely responding to a tactile stimulus; rather, it could potentially be matching perceived inertial information against a prior expectation derived from the object's visual size.

Across both experiments, the most powerful predictor of the crows' final choice was the weight ratio of two walnuts. This finding is consistent with the idea that crows evaluate physical stimuli through proportional processing, a principle fundamentally described by Weber's Law. According to this psychophysical principle, the perceived difference between two stimuli is proportional to their magnitude, a mechanism that has been observed in various animals across domains such as number, size, and weight (Akre and Johnsen 2014; Eckert et al. 2018). Our results align with captive studies on jungle crows and carrion crows, which have demonstrated proportional discrimination in numerical tasks (Bogale et al. 2011, 2014; Ditz and Nieder 2016). By observing this in a naturalistic foraging context, we provide evidence suggesting that the crows' decision‐making system may be tuned to relative, rather than absolute, differences between rewards. However, it should be noted that the use of natural walnuts in this study imposed inherent constraints on the variation of size and weight ratios. To more precisely define the underlying perceptual mechanisms, such as the Weber fraction or the specific discrimination threshold, rigorous laboratory‐based research remains essential.

Our study also demonstrated a significant effect of the study site in Experiment 1. Crows at the River and South Campus exhibited higher accuracy in both initial contact with and final retrieval of the larger walnut compared to the reference site. Because this difference could not be explained by regional variations (Uenohara vs. Sendai) or group composition (territorial pairs vs. non‐breeding juveniles), the precise underlying reason remains unclear. Nevertheless, this finding suggests that the ability to discriminate and express preferences based on the physical properties of prey is a flexible trait that can vary according to individual experiences and local environmental context.

However, the interpretation of these site‐level variations and individual flexibility must be considered alongside a methodological limitation of our field setup. Chief among these is the absence of individual color‐banding, which introduces the potential risk of pseudoreplication through the repeated testing of the same individuals. We cannot entirely rule out the possibility that certain wide‐ranging individuals contributed multiple data points in flock‐dominated areas. Future studies utilizing uniquely marked populations will be critical to explicitly parse individual‐level variance from population‐level heuristics in wild corvids.

Furthermore, the cognitive performance documented here ultimately reflects “realized cognitive ability” under complex environmental conditions. Quantitative decision‐making in the wild is modulated by an individual's behavioral decisions, such as habitat choice, primary prey selection, micro‐site selection, and propensity to join social groups, as well as interactions with fluctuating ecological and social dynamics (Miller et al. 2014; Morand‐Ferron et al. 2016; Benson‐Amram et al. 2017). To fully evaluate how the multimodal decision‐making processes observed in this study translate into actual fitness consequences, such as energy intake efficiency, survival, and reproductive success, future research should integrate controlled laboratory tasks with longitudinal tracking of ecological trade‐offs in marked wild populations.

In conclusion, our findings demonstrate that crows optimize their foraging decisions through a flexible, multimodal strategy that integrates visual and haptic information. This process of relative evaluation, governed by proportional processing, ensures that their energetic investment is directed toward the most profitable resources. The present study contributes to our understanding of the physical cognition and adaptive decision‐making processes in wild corvids.

## Author Contributions

**Torito Hamano:** conceptualization (lead), data curation (equal), formal analysis (equal), investigation (lead), methodology (equal), visualization (equal), writing – original draft (lead), writing – review and editing (equal). **Yurika Mitsuhashi:** data curation (equal), formal analysis (equal), investigation (equal). **Noriko Kondo:** conceptualization (equal), writing – review and editing (equal). **Masaki Shimada:** conceptualization (equal), writing – review and editing (equal). **Akitsugu Konno:** conceptualization (lead), data curation (equal), formal analysis (equal), investigation (supporting), methodology (lead), project administration (lead), visualization (equal), writing – original draft (equal), writing – review and editing (lead).

## Ethics Statement

The present study protocol was approved by the Animal Experiment Committee at Teikyo University of Science (Approval Nos. 21C039, 22C035). The protocol adhered to the guidelines for the treatment of animals in behavioral research and teaching established by the Animal Behavior Society and the Association for the Study of Animal Behavior: (Buchanan et al. 2020).

## Conflicts of Interest

The authors declare no conflicts of interest.

## Supporting information


**Figure S1:** A crow choosing between walnuts in Experiment 1 (Photo credit: Hiroya Minakuchi).

**Figure S2:** Preparation of experimental stimuli (Empty and Filled walnuts) used in Experiment 2.

**Table S1:** Summary of walnut selection across four study sites in Experiment 1 (size preference).
**Table S2:** Summary of walnut selection across two study sites in Experiment 2 (content preference).

## Data Availability

The data supporting the findings of this study are currently uploaded as Supporting Information for the review process. Upon acceptance, the data will be deposited in the Dryad Digital Repository (10.5061/dryad.tb2rbp0k1).
