# Supplementary material for: Wild Carrion Crows Choose Walnuts Based on Visual and Weight Cues
Source: Ecol Evol. 2026 Oct 20;16(10):e74554. doi: 10.1002/ece3.74554 (PMC13681255; doi:10.1002/ece3.74554)
Supplement: Supplementary file 1 [file ECE3-16-e74554-s002.docx]

## **Appendix**

### Table S1. Summary of walnut selection across four study sites in Experiment 1 (Size Preference).

| Study Sites | Large Nut First-Contact Rate | Large Nut Retrieval Rate |
| --- | --- | --- |
| University (Uenohara) | 44/77 (57.1 %) | 57/77 (74.0 %) |
| River (Uenohara) | 65/85 (76.5 %) | 76/85 (89.4 %) |
| North Campus (Sendai) | 16/20 (80.0 %) | 16/20 (80.0 %) |
| South Campus (Sendai) | 36/59 (61.0 %) | 46/59 (78.0 %) |
| Total | 161/241 (66.8 %) | 195/241 (80.9 %) |

### Table S2. Summary of walnut selection across two study sites in Experiment 2 (Content Preference).

| Study Sites | Filled Nut First-Contact Rate | Filled Nut Retrieval Rate |
| --- | --- | --- |
| University (Uenohara) | 23/40 (57.5 %) | 28/40 (70.0 %) |
| River (Uenohara) | 24/40 (60.0 %) | 32/40 (80.0 %) |
| Total | 47/80 (58.8 %) | 60/80 (75.0 %) |

Figure S1. A crow choosing between walnuts in Experiment 1 (Photo credit: Hiroya Minakuchi).

Figure S2. Preparation of experimental stimuli (Empty and Filled walnuts) used in Experiment 2.

**a) Empty:** A natural walnut was split into two halves, and the kernel was completely removed. The empty shells were then glued back together to create an "Empty" stimulus that appeared intact from the outside but lacked internal mass.

**b) Filled):** Similarly, a natural walnut was split, and the internal kernel was either kept intact or replaced/adjusted to ensure it was "Filled." The two halves were then glued back together.

These procedures ensured that the visual appearance of both stimuli was nearly identical, forcing the crows to rely on weight-related or acoustic cues rather than visual information for discrimination.
